# Draft Genome Sequences of Type Strains *Bacillus drentensis* DSM 15600^T^ and *Bacillus novalis* DSM 15603^T^

**DOI:** 10.1128/genomeA.01423-16

**Published:** 2016-12-15

**Authors:** Bo Liu, Guo-Hong Liu, Yu-jing Zhu, Jie-Ping Wang, Jian-Mei Che, Qian-Qian Chen, Zheng Chen

**Affiliations:** Agricultural Bio-Resources Research Institute, Fujian Academy of Agricultural Sciences, Fuzhou, China

## Abstract

Here, we report the draft genome sequences of *Bacillus drentensis* DSM 15600^T^ and *Bacillus novalis* DSM 15603^T^ with 5,305,306 bp and 5,667,584 bp, respectively, which will provide useful information for the functional gene mining and application of these two species. The average DNA G+C contents were 38.91% and 40.01%, respectively.

## GENOME ANNOUNCEMENT

Type strains *Bacillus drentensis* DSM 15600^T^ and *Bacillus novalis* DSM 15603^T^ are Gram-positive, spore-forming, and aerobic bacteria, isolated from the soil of several disused hay fields in the Drentse A agricultural research area (the Netherlands). As a result of the recent decrease in the cost of genomic sequencing, it has been proposed that whole-genome sequencing information be combined with the main phenotypic characteristics as a polyphasic approach strategy (taxono-genomics) to describe new bacterial taxa (1–4). In this study, a high-quality genome sequence of *B. drentensis* DSM 15600^T^ was sequenced, which may promote research in the genomic taxonomy of the *Bacillus*-like bacteria.

The genomes of *B. drentensis* DSM 15600^T^ and *B. novalis* DSM 15603^T^ were sequenced with massively parallel sequencing (MPS) Illumina technology. Two DNA libraries were constructed: a paired-end library with an insert size of 500 bp and a mate-pair library with an insert size of 5 kb. The 500-bp library and the 5-kb library were sequenced using an Illumina HiSeq 2500 by PE125 strategy. Library construction and sequencing were performed at the Beijing Novogene Bioinformatics Technology Co., Ltd. Quality control of both paired-end and mate-pair reads were performed using an in-house program. After this step, Illumina PCR adapter reads and low-quality reads were filtered. The filtered reads were assembled by SOAPdenovo (5, 6) to generate scaffolds. All reads were used for further gap closure. Through the data assembly, 5,305,306 bp within three scaffolds and 5,668,192 bp within two scaffolds were obtained, and the scaffold *N*_50_ values were 5,303,701 bp and 5,667,584 bp, respectively, for *B. drentensis* DSM 15600^T^ and *B. novalis* DSM 15603^T^. The longest and shortest scaffolds of these two species were 5,303,701 bp and 689 bp and 5,667,584 bp and 608 bp, respectively.

For the genome assemblies of these two species, gene prediction was performed with GeneMarkS (7). Transfer RNA (tRNA) genes were predicted with tRNAscan-SE (8), rRNA genes were predicted with rRNAmmer (9) and short RNAs (sRNAs) were predicted by BLAST against the Rfam (10) database. PHAST (11) was used for prophage prediction and CRISPRFinder (12) was used for clustered regularly interspaced short palindromic repeat (CRISPR) identification. Totals of 5,516 and 5,986 genes of *B. drentensis* DSM 15600^T^ and *B. novalis* DSM 15603^T^ were predicted, including 5,337 and 5,827 coding sequences (CDS), respectively, and four sRNAs, 125 tRNAs, 50 rRNAs (17 5S, 16 16S, and 17 23S) and five sRNAs, 118 tRNAs, 36 rRNA (13 5S, 11 16S, and 12 23S), respectively. The average DNA G+C contents were 38.91% and 40.01%, respectively.

### Accession number(s).

These whole-genome shotgun projects for *B. drentensis* DSM 15600^T^ and *B. novalis* DSM 15603^T^ have been deposited at DDBJ/EMBL/GenBank under the accession numbers LUUU00000000 and LUUR00000000, respectively. The versions described in this paper are versions LUUU00000000.1 and LUUR00000000.1.
